# Clinical Use and Application of Neutropenic Diets for Patients With Cancer: A Cross‐Sectional Survey of Dietitians in Australian Hospitals

**DOI:** 10.1111/jhn.70228

**Published:** 2026-03-12

**Authors:** Trinity Gulliver, Melissa Hewett, Panagiotis Konstantopoulos, Lisa Tran, Evangeline Mantzioris

**Affiliations:** ^1^ Allied Health and Human Performance Adelaide University Adelaide South Australia Australia; ^2^ Department of Nutrition & Dietetics Royal Adelaide Hospital Adelaide South Australia Australia; ^3^ Alliance for Research in Exercise, Nutrition & Activity (ARENA) Adelaide University Adelaide South Australia Australia

**Keywords:** Australia, cancer, diet, food safety, hospitals

## Abstract

**Background:**

The neutropenic diet (ND) has historically been prescribed to reduce infection risk in immunocompromised patients with cancer, despite limited supporting evidence. This study aimed to evaluate current practices surrounding ND use in Australian hospitals.

**Methods:**

An online survey was distributed to dietitians working with hospitalised patients with cancer. The survey explored dietary practices, foods restricted, initiation and discontinuation criteria, and dietitians' perspectives on ND use.

**Results:**

Overall, 56% of responding dietitians reported that their hospitals prescribe an ND. Among these, there was broad consistency in the restriction of approximately 85% of foods, though considerable variation existed regarding criteria for ND initiation and discontinuation. Dietitians' personal views on ND use generally aligned with their hospital's practice (87.0%).

**Conclusions:**

More than half of Australian hospitals surveyed continue to prescribe an ND, with general consistency in food restrictions but variation in initiation and discontinuation practices. These findings highlight the need for institutional review of ND practices and support the development of evidence‐based food safety education to better guide dietary care for patients with cancer.

## Summary

- Over half (56%) of dietitians in Australia report continued use of neutropenic diets in hospitals.
- Neutropenic diet protocols showed high consistency (85%) in permitted foods.
- Given no evidence of reduced infection or mortality, hospitals should review neutropenic diet policies.

## 1 Introduction

People with cancer, particularly those receiving immunosuppressive chemotherapy, are at risk of neutropenia, defined as an absolute neutrophil count < 1.5 × 10⁹/L, which increases susceptibility to infection and other adverse effects associated with intensive chemotherapy [1, 2]. Historically, the neutropenic diet (ND) has been recommended to reduce infection risk by restricting foods with higher bacterial loads, such as raw fruits and vegetables, undercooked meat, fish, eggs, and unpasteurised dairy [3, 4]. However, the ND is not standardised, with restricted foods varying across dietitians and hospitals [3].

An alternative approach, the food safety diet (FSD), is more liberal than the ND, allowing a wider variety of foods while restricting only a small number of high‐risk items such as undercooked meat, fish, eggs, and unpasteurised dairy, with the emphasis placed on safe food hygiene, preparation, cooking, and storage [4]. Current evidence indicates that the ND does not reduce infection or mortality compared with the FSD [5, 6, 7] and European Society for Clinical Nutrition and Metabolism (ESPEN) guidelines do not recommend its use in cancer care. Furthermore, the ND may compromise dietary variety and quality of life, particularly in paediatric patients [8, 9].

Despite this, the ND remains common for patients with cancer, with 50%–80% of hospitals in Europe [10, 11, 12, 13], the United States [14], and China [15] reporting its use.

In Australia, the prevalence of ND use is unknown, although anecdotal evidence suggests it remains in practice. No study has assessed dietitians' use of the ND in this context. This study therefore aimed to determine the extent to which Australian hospital dietitians provide or recommend the ND (or components of it) to patients with cancer. Secondary aims were to examine dietitians' provision of food safety education, use of nutritional screening, knowledge of the ND, and opinions regarding its appropriateness of the ND for patients with cancer.

## 2 Methods

A cross‐sectional online study was conducted among Australian dietitians working with people with cancer (current or within the past 12 months) who could complete an English‐language survey.

Recruitment was carried out through multiple channels between November 2021 and August 2023. Dietitians were invited to participate via two issues of the Dietitian Connection newsletter (22 November 2021 and 19 June 2022) and targeted social media posts by the investigators on X (formerly Twitter) and LinkedIn. Hospitals offering cancer services, (as identified via the Australian Institute of Health and Welfare) [16] were contacted by email, online enquiry forms, or telephone to reach the relevant department (e.g., cancer services, dietetics, or allied health). If no response was received, follow‐up phone contact was attempted two to three times.

The survey was developed and distributed using REDCap (Nashville, TN, United States). A link to the survey, accompanied by a participant information sheet outlining the study protocol and risks, was shared via newsletters, social media, and direct hospital emails. During recruitment, participants were informed that the survey related to dietary recommendations for patients with cancer; only upon completion did the specific focus on the ND become clear. The study was approved by the University of South Australia Human Research Ethics Committee (HREC 203955; 7 September 2021) and conducted in accordance with the Declaration of Helsinki. Participation was voluntary, confidential, and required acknowledgement of informed consent.

The survey was developed by the authorship team, comprising a master's student, a food science specialist, an academic dietitian with prior clinical experience, and two clinical dietitians working in cancer services. It was constructed in REDCap and used branched logic to tailor questions to participants' responses. Survey development was iterative, with revisions made to optimise content and flow. Prior to launch, five external clinical dietitians piloted the survey and provided feedback on usability and clarity, which informed final modifications.

The survey comprised five sections (1): Participant characteristics—demographics, workplace, and patient groups consulted (2); Dietary restrictions—foods commonly restricted for patients with cancer (haematological and/or oncological cancers), including hospital, takeaway, and home‐prepared foods (3); Patient education—provision of food safety education (4); Nutritional screening—use of screening tools, timing, and responsible staff; and (5) The ND—use, indications, and awareness. In the final section, respondents were also provided with an abstract and link to a recent systematic review on the ND and asked: “Based on your own opinion and knowledge of the evidence base, how do you think we should proceed with the use of neutropenic diets for cancer patients?”

Data was extracted from REDCap into Microsoft Excel (Microsoft Corporation, WA, US) and analysed in IBM SPSS (Armonk, NY, US). Responses were deidentified to ensure confidentiality. Descriptive statistics (frequencies and percentages) were calculated for multiple‐choice items. As the study was exploratory and designed to characterise current practice rather than assess associations or group differences, no inferential statistical testing was undertaken. Extended responses were thematically analysed in Excel, with four authors (EM, TG, MH, PK) independently reviewing data and discrepancies resolved through discussion among three authors (EM, TG and MH).

## 3 Results

The survey was available online from November 2021 to August 2023. This extended data collection period was due to the low response rate, likely influenced by the impact of COVID‐19 on hospital services and dietitians' workloads. A total of 149 Australian hospitals were contacted, with 48 responses received from dietitians. These responses represented 39 hospitals (26.1% response rate), including nine hospitals where two dietitians participated.

Demographics: 31 respondents completed the survey in full. All participating dietitians were women; half had more than 10 years of professional experience. Most respondents (81.3%) were employed in public hospitals. All dietitians reported working with oncology patients, and 70.8% also managed patients with haematological malignancies.

Characteristics of responding dietitians and their hospitals are presented in Table 1.

**Table 1:** Demographic characteristics of dietitians surveyed and the hospitals they work in.

Characteristics	Number (%) (*n* = 48)
Gender	
Woman	48 (100.0)
Years qualified as dietitian	
< 2	5 (10.4)
2–5	11 (22.9)
6–10	8 (16.7)
> 10	24 (50.0)
Years practicing as dietitian	
< 2	6 (12.5)
2–5	10 (20.8)
6–10	8 (16.7)
> 10	24 (50.0)
Dietitians consulting haematology or oncology patients	
Haematology	34 (70.8)
Oncology	48 (100.0)
Australian state hospital resides	
ACT	2 (4.2)
NSW	12 (25)
NT	1 (2.1)
QLD	5 (10.4)
SA	11 (22.9)
TAS	3 (6.3)
VIC	8 (16.7)
WA	6 (12.5)
Public/private hospital	
Public	39 (81.3)
Private	9 (18.8)
No. Total beds	
< 100	4 (8.3)
100–299	10 (2.08)
300–499	14 (29.2)
500–699	11 (22.9)
700–899	6 (12.5)
> 900	3 (6.3)
No. Cancer beds	
< 10	16 (33.3)
10–29	10 (20.8)
30–49	9 (18.8)
50–69	9 (18.8)
70–89	3 (6.3)
> 90	1 (2.1)
No. Haematology beds	
< 10	22 (45.8)
10–29	20 (41.7)
30–49	5 (10.4)
50–69	1 (2.1)
No. Neutropenic inpatients	
< 10	31 (64.6)
10–29	15 (31.3)
30–49	2 (4.2)
Hospital provides stem cell transplant	
Autologous only	7 (14.6)
Autologous & Allogeneic	12 (25.0)
No	29 (60.4)

Dietary restrictions: Overall, 56% of surveyed hospitals reported using an ND for patients with cancer. ND use was most common in larger hospitals, with 80% of hospitals with > 500 total beds adopting the practice. By contrast, ND use was similar in hospitals with more than 30 oncology beds (55.5% prescribing, 33.3% not prescribing) and haematology beds (11.1% prescribing, 13.3% not prescribing), and use was broadly comparable between public (62.9%) and private hospitals (71.4%). Years of clinical practice experience appeared to have minimal influence on overall prescription of the ND diet. Similar proportions of dietitians with 6–10 years of experience reported prescribing and not prescribing the ND diet (*n* = 4 in each group), as did those with more than 10 years of experience (*n* = 13 prescribing; *n* = 11 not prescribing). Greater variability was observed among dietitians with fewer than 5 years of experience. All dietitians with less than 2 years of experience reported prescribing the ND diet (*n* = 5), whereas among those with 2–5 years of experience, four reported prescribing and seven reported not prescribing.

Dietitians were asked to indicate which foods were commonly restricted for patients with haematological and/or oncological cancers, irrespective of whether the diet was labelled as a ND. Approximately half of respondents (*n* = 23 dietitians from 21 hospitals) provided data, presented in Table 2. The table reports the number of dietitians restricting each food and compares restrictions between those prescribing and not prescribing a ND. ND use was classified based on reported food restrictions, the terminology used to describe the diet, and responses to the direct survey item on ND use. Among dietitians not prescribing a ND, the most frequently restricted foods were unpasteurised dairy products, raw or partially cooked red meat, fish and shellfish, pâté, and food from buffets/smorgasbords.

**Table 2:** Foods restricted for inpatient oncology and haematology wards in hospitals implementing a neutropenic diet versus hospitals not implementing a neutropenic diet^a^.

	Haematology				Oncology			
	Hospitals with ND (*n* = 18)		Hospitals without ND (*n* = 4)		Hospitals with ND (*n* = 12)		Hospitals without ND (*n* = 4)	
	Restricted	Not restricted	Restricted	Not restricted	Restricted	Not restricted	Restricted	Not restricted
	*n* (%)	*n* (%)	*n* (%)	*n* (%)	*n* (%)	*n* (%)	*n* (%)	*n* (%)
Fruit + vegetables								
Thick skinned fruit	1 (5.6)	17 (94.4)	0 (0.0)	4 (100.0)	1 (8.3)	11 (91.7)	0 (0.0)	4 (100.0)
Thin skinned fruit (unwashed)	13 (72.2)	5 (27.8)	1 (25.0)	3 (75.0)	7 (58.3)	5 (41.7)	1 (25.0)	3 (75.0)
Thin skinned fruit (washed)	3 (16.7)	15 (83.3)	0 (0.0)	4 (100.0)	3 (25.0)	9 (75.0)	0 (0.0)	4 (100.0)
Berries (unwashed)	13 (72.2)	5 (27.8)	1 (25.0)	3 (75.0)	8 (66.7)	4 (33.3)	2 (50.0)	2 (50.0)
Berries (washed)	8 (44.4)	10 (55.6)	0 (0.0)	4 (100.0)	4 (33.3)	8 (66.7)	0 (0.0)	4 (100.0)
Dried fruit	4 (22.2)	14 (77.8)	0 (0.0)	4 (100.0)	2 (16.7)	10 (83.3)	0 (0.0)	4 (100.0)
Fruit juice (pasteurised)	0 (0.0)	18 (100)	0 (0.0)	4 (100.0)	0 (0.0)	12 (100.0)	0 (0.0)	4 (100.0)
Fruit juice (unpasteurised)	14 (77.8)	4 (22.2)	1 (25.0)	3 (75.0)	8 (66.7)	4 (33.3)	2 (50.0)	2 (50.0)
Fruit juice (freshly squeezed)	9 (50.0)	9 (50.0)	1 (25.0)	3 (75.0)	4 (33.3)	8 (66.7)	1 (25.0)	3 (75.0)
Raw vegetables (unwashed)	16 (88.9)	2 (11.1)	1 (25.0)	3 (75.0)	8 (66.7)	4 (33.3)	2 (50.0)	2 (50.0)
Raw vegetables (washed)	5 (27.8)	13 (72.2)	0 (0.0)	4 (100.0)	4 (33.3)	8 (66.7)	0 (0.0)	4 (100.0)
Cooked vegetables	0 (0.0)	18 (100)	0 (0.0)	4 (100.0)	0 (0.0)	12 (100.0)	0 (0.0)	4 (100.0)
Dairy								
Yoghurt	2 (11.1)	16 (88.9)	0 (0.0)	4 (100.0)	0 (0.0)	12 (100.0)	0 (0.0)	4 (100.0)
Unpasteurised dairy	16 (88.9)	2 (11.1)	2 (50.0)	2 (50.0)	9 (75.0)	3 (25.0)	2 (50.0)	2 (50.0)
Soft cheese	16 (88.9)	2 (11.1)	1 (25.0)	3 (75.0)	9 (66.7)	3 (33.3)	2 (50.0)	2 (50.0)
Hard cheese	0 (0)	18 (100)	0 (0.0)	4 (100.0)	0 (0.0)	12 (100.0)	0 (0.0)	4 (100.0)
Cream	2 (11.1)	16 (88.9)	0 (0.0)	4 (100.0)	2 (16.7)	10 (83.3)	0 (0.0)	4 (100.0)
Dairy alternatives	0 (0)	18 (100)	0 (0.0)	4 (100.0)	0 (0.0)	12 (100.0)	0 (0.0)	4 (100.0)
Protein foods								
Red meat (well cooked)	0 (0.0)	18 (100)	0 (0.0)	4 (100.0)	0 (0.0)	12 (100.0)	0 (0.0)	4 (100.0)
Red meat (partially cooked)	17 (94.4)	1 (5.6)	2 (50.0)	2 (50.0)	10 (83.3)	2 (16.7)	1 (25.0)	3 (75.0)
Chilled well‐cooked meats	8 (44.4)	10 (55.6)	0 (0.0)	4 (100.0)	5 (41.7)	7 (58.3)	0 (0.0)	4 (100.0)
Deli meats	13 (72.2)	5 (27.8)	1 (25.0)	3 (75.0)	8 (66.7)	4 (33.3)	1 (25.0)	3 (75.0)
Pâté	15 (83.3)	3 (16.7)	2 (50.0)	2 (50.0)	9 (75.0)	3 (25.0)	2 (50.0)	2 (50.0)
Fish (well cooked)	0 (0.0)	18 (100)	0 (0.0)	4 (100.0)	0 (0.0)	12 (100.0)	0 (0.0)	4 (100.0)
Fish (raw/partially cooked)	17 (94.4)	1 (5.6)	2 (50.0)	2 (50.0)	10 (83.3)	2 (16.7)	2 (50.0)	2 (50.0)
Fish (smoked/cured)	13 (72.2)	5 (27.8)	1 (25.0)	3 (75.0)	7 (58.3)	5 (41.7)	2 (50.0)	2 (50.0)
Shellfish (well cooked)	5 (27.8)	13 (72.2)	0 (0.0)	4 (100.0)	1 (8.3)	11 (91.7)	0 (0.0)	4 (100.0)
Shellfish (raw/partially cooked)	17 (94.4)	1 (5.6)	2 (50.0)	2 (50.0)	10 (83.3)	2 (16.7)	2 (50.0)	2 (50.0)
Poultry	0 (0.0)	18 (100)	0 (0.0)	4 (100.0)	0 (0.0)	12 (100.0)	0 (0.0)	4 (100.0)
Egg (well cooked)	0 (0.0)	18 (100)	0 (0.0)	4 (100.0)	0 (0.0)	12 (100.0)	0 (0.0)	4 (100.0)
Egg (partially cooked)	17 (94.4)	1 (5.6)	2 (50.0)	2 (50.0)	9 (66.7)	3 (33.3)	2 (50.0)	2 (50.0)
Nuts (raw)	9 (50.0)	9 (50.0)	1 (25.0)	3 (75.0)	5 (41.7)	7 (58.3)	0 (0.0)	4 (100.0)
Nuts (roasted)	2 (7.4)	16 (88.9)	0 (0.0)	4 (100.0)	2 (16.7)	10 (83.3)	0 (0.0)	4 (100.0)
Nut spreads	2 (7.4)	16 (88.9)	0 (0.0)	4 (100.0)	2 (16.7)	10 (83.3)	0 (0.0)	4 (100.0)
Vegan/vegetarian meat alternatives	1 (3.7)	17 (94.4)	0 (0.0)	4 (100.0)	0 (0.0)	12 (100.0)	0 (0.0)	4 (100.0)
Cereals								
Raw cereals	6 (33.3)	12 (66.7)	0 (0.0)	4 (100.0)	3 (25.0)	9 (75.0)	0 (0.0)	4 (100.0)
Commercial cereals	0 (0.0)	18 (100)	0 (0.0)	4 (100.0)	0 (0.0)	12 (100.0)	0 (0.0)	4 (100.0)
Seeded bread	1 (3.7)	17 (94.4)	0 (0.0)	4 (100.0)	0 (0.0)	12 (100.0)	0 (0.0)	4 (100.0)
Beverages								
Tap water	4 (22.2)	14 (77.8)	0 (0.0)	4 (100.0)	2 (16.7)	10 (83.3)	0 (0.0)	4 (100.0)
Filtered water	1 (3.7)	17 (94.4)	0 (0.0)	4 (100.0)	0 (0.0)	12 (100.0)	0 (0.0)	4 (100.0)
Bottled mineral water	2 (7.4)	16 (88.9)	0 (0.0)	4 (100.0)	2 (16.7)	10 (83.3)	0 (0.0)	4 (100.0)
Bottled sparkling water	2 (7.4)	16 (88.9)	0 (0.0)	4 (100.0)	2 (16.7)	10 (83.3)	0 (0.0)	4 (100.0)
Herbal/flavoured tea	3 (16.7)	15 (83.3)	0 (0.0)	4 (100.0)	1 (8.3)	11 (91.7)	0 (0.0)	4 (100.0)
Alcohol	9 (50.0)	9 (50.0)	0 (0.0)	4 (100.0)	4 (33.3)	8 (66.7)	1 (25.0)	3 (75.0)
Kombucha	10 (55.6)	8 (44.4)	0 (0.0)	4 (100.0)	4 (33.3)	8 (66.7)	1 (25.0)	3 (75.0)
Other								
Raw herbs/spices	5 (27.8)	13 (72.2)	1 (25.0)	3 (75.0)	3 (25.0)	9 (75.0)	2 (50.0)	2 (50.0)
Raw honey	9 (50.0)	9 (50.0)	1 (25.0)	3 (75.0)	5 (41.7)	7 (58.3)	2 (50.0)	2 (50.0)
Infant formula	2 (11.1)	16 (88.9)	0 (0.0)	4 (100.0)	1 (8.3)	11 (91.7)	0 (0.0)	4 (100.0)
Buffet/smorgasbord	14 (77.8)	4 (22.2)	2 (50.0)	2 (50.0)	8 (66.7)	4 (33.3)	1 (25.0)	3 (75.0)
Large/multiple‐use containers	9 (50.0)	9 (50.0)	1 (25.0)	3 (75.0)	3 (25.0)	9 (75.0)	1 (25.0)	3 (75.0)

	Hospitals with ND (*n* = 15) (%)		Hospitals without ND (*n* = 9) (%)		Hospitals with ND (*n* = 21) (%)		Hospitals without ND (*n* = 12) (%)	
	Restricted	Not restricted	Restricted	Not restricted	Restricted	Not restricted	Restricted	Not restricted
	*n* (%)	*n* (%)	*n* (%)	*n* (%)	*n* (%)	*n* (%)	*n* (%)	*n* (%)
Takeaway	4 (26.7)	11 (73.3)	0 (0.0)	9 (100)	2 (9.5)	19 (90.5)	0 (0.0)	12 (100.0)
Meal delivery	5 (33.3)	10 (66.7)	1 (11.1)	8 (88.9)	2 (9.5)	19 (90.5)	1 (8.3)	11 (91.7)

	Hospitals with ND (*n* = 13) (%)		Hospitals without ND (*n* = 9) (%)		Hospitals with ND (*n* = 20) (%)		Hospitals without ND (*n* = 12) (%)	
	Restricted	Not restricted	Restricted	Not restricted	Restricted	Not restricted	Restricted	Not restricted
	*n* (%)	*n* (%)	*n* (%)	*n* (%)	*n* (%)	*n* (%)	*n* (%)	*n* (%)
Food from home	1 (7.7)	12 (92.3)	0 (0.0)	9 (100.0)	0 (0.0)	20 (100.0)	0 (0.0)	12 (100.0)

a Results from dietitians who either did not identify their protocol as a neutropenic (ND) diet or provided insufficient detail for classification have been omitted. Use of ND was determined by (a) the set of restricted foods listed and (b) the terminology used by the dietitian in the survey.

Dietitians prescribing a ND for patients with cancer reported more extensive restrictions. Foods restricted by more than two‐thirds included: unpasteurised fruit juice and dairy products, unwashed raw vegetables and berries, raw/partially cooked fish and shellfish, partially cooked eggs and red meat, deli meats, pâté, soft cheeses, and food from buffets/smorgasbords. For patients with haematological cancers, further restrictions applied, with > 66.7% of dietitians excluding unwashed thin‐skinned fruit and smoked/cured fish.

Certain foods were restricted by approximately half of dietitians, for the haematology patients on a ND, these included washed berries, freshly squeezed fruit juice, alcohol, kombucha, and foods from large/multi‐use containers (e.g., mayonnaise). For oncology patients, unwashed thin‐skinned fruit and smoked/cured fish were reported, while across all cancer patients, chilled well‐cooked meats, raw nuts, and raw honey were restricted by about half. Conversely, some foods were consistently permitted across both groups, including pasteurised fruit juice, cooked vegetables, hard cheese, dairy alternatives, well‐cooked red meat, well‐cooked fish, poultry, well‐cooked eggs, and commercial cereals. Additionally, yoghurt, plant‐based meat alternatives, and filtered water were generally not restricted for oncology patients.

The majority of dietitians permitted patients to consume takeaway food (83.3% of dietitians prescribing an ND; 100% of dietitians not prescribing an ND) and food from commercial meal delivery services such as UberEats, MenuLog, and DoorDash (80.5% and 90.5%, respectively). However, some dietitians placed restrictions on the types of foods that could be selected, with fewer restrictions typically applied to oncology patients compared with haematology patients (Table 2).

Several dietitians elaborated on their approach. One, working with both haematology and oncology patients, noted:

*“If [a] patient is neutropenic (neutrophils* <*1.0) then they would be advised to only order freshly cooked hot food that doesn't include any restricted foods and that it is consumed immediately whilst hot*” (P007).

Another dietitian highlighted patient autonomy:

“*Patients are educated [about] the risks by dietitian and ward staff. [Patients] can still choose to include these foods as they are aware [of] the risk, but we cannot stop them from including such foods as they are voluntary patients*” (P044).

A third dietitian emphasised specific exclusions for haematology patients:

“*Avoid salad containing options, sushi, soft serve*” (P046).

Among the eight dietitians who reported placing restrictions on takeaway/meal delivery, half observed that these restrictions were followed only some of the time.

Most dietitians also permitted both haematology and oncology patients to consume food brought into hospital by family or friends (Table 2). Nonetheless, restrictions were more common for patients prescribed to follow a ND (75.0% of haematology and 30.0% of oncology patients) compared with those not prescribed a ND (22.2% and 16.7%, respectively). Ten dietitians provided further detail on the types of restrictions applied. Four emphasised patient and/or family food safety education, three noted the need to align foods with the diet prescribed to the patient (i.e., excluding restricted items), and one reported that any food brought in must be discarded if not consumed within 24 h. Practices around reheating food varied: while two dietitians permitted chilled food to be reheated in the hospital, another prohibited food requiring reheating altogether.

Patient education: Most dietitians (80% using a ND, 66.7% not using a ND) reported not providing food safety education to all patients as standard protocol, instead targeting education to patients meeting specific criteria. Neutropenia was the most common criterion (62.5%), followed by chemotherapy (20.8%), stem cell transplantation (20.8%), and immunocompromised status (8.3%). Education was usually delivered face‐to‐face with supplementary take‐home resources. Nutrition Education Materials Online (NEMO) [17] food safety sheets were used by 20.7% (*n* = 6) of dietitians, other reported resources included Food Standards Australia New Zealand (FSANZ) (6.9%), ESPEN (3.4%) and Cancer Council Australia (3.4%).

Reported education topics included restriction of high‐risk foods (57.7%), safe preparation and storage (50.0%), management of leftovers and reheating (15.4%), and hygiene practices (11.6%). Only one dietitian specifically reported providing education on the ND.

Nutrition Screening: All dietitians, irrespective of ND use, reported that patients with cancer were routinely screened for nutritional status. The most commonly used tool was the Malnutrition Screening Tool (MST; 75.0% ND, 83.3% non‐ND), followed by the Patient‐Generated Subjective Global Assessment (PG‐SGA) and the Malnutrition Universal Screening Tool (MUST). Screening was typically undertaken by nursing staff and most often occurred at admission, though it was reported at various points during the hospital stay (Table 3). No differences in screening practices were observed between hospitals that prescribed a ND and those that did not.

**Table 3:** Nutritional‐status screening of patients in hospitals implementing the neutropenic diet versus patients in hospitals not implementing neutropenic diet.

	Hospitals with ND (*n* = 20) (%)	Hospitals without ND (*n* = 12) (%)
Patients screened		
Yes	20 (100)	12 (100)
No	0 (0)	0 (0)
Screening tool^a^		
PG‐SGA	6 (30.0)	1 (8.3)
MST	15 (75.0)	10 (83.3)
MUST	5 (25.0)	1 (8.3)
GLIM	1 (5.0)	0 (0)
PNST	0 (0)	1 (8.3)
Role of person who screened^a^		
Nurse	17 (85.0)	12 (100)
Dietitian	7 (35.0)	3 (25.0)
Allied health assistant	6 (30.0)	3 (25.0)
Nutrition assistant	4 (20.0)	1 (8.3)
Time of screening^a^		
Initial assessment	7 (35.0)	1 (8.3)
Admission	19 (95.0)	12 (100)
Any time	6 (30.0)	3 (25.0)
Weekly	3 (11.1)	3 (25.0)
Start of treatment	3 (11.1)	3 (25.0)
Discharge	2 (10.0)	0 (0)

*Note:* a—more than one response was allowed for this question.

Abbreviations: GLIM, global leadership initiative on malnutrition; MST, malnutrition screening tool; MUST, malnutrition universal screening tool; PG‐SGA, patient generated subjective global assessment; PNST, paediatric nutrition screening tool.

ND—use, indications and awareness: All dietitians, except one, reported prior awareness of the ND, and just over half had received education on the ND during university training. The ND was initiated by most dietitians based on neutrophil count (42.1%)—although the specific level of neutrophils at which initiation occurred differed. Individual patient assessment (36.8% of dietitians) and neutrophil count (26.3% of dietitians) were the most frequent rationale for discontinuing the ND for patients, similarly to ND initiation, the specific level of neutrophils at which discontinuation occurred differed. Among those prescribing the ND, the most common trigger for implementation was neutrophil count, although the thresholds used varied. Three dietitians reported using two to three combined criterion to guide initiation, though it was unclear whether all factors were applied simultaneously or selectively across patients. Most dietitians discontinued the ND on an individual basis, with 69.2% reporting that nutritional status did not influence decisions to initiate or discontinue the diet (Table 4). No dietitians recommended continuing the ND post‐discharge; instead, patients were advised to follow a FSD (58.4%) or received no dietary recommendations (41.6%).

**Table 4:** Reasons for initiating, discontinuing and applying the neutropenic diet.

Initiation	(*n* = 18) (%)
Neutrophil count	8 (42.1)
< 0.5 × 10^9^/L	2 (25.0)
< 1.0 × 10^9^/L	4 (50.0)
< 2.0 × 10^9^/L	1 (12.5)
Unsure	1 (12.5)
Admission	3 (15.8)
Individual basis	4 (31.1)
Treatment commencement	3 (15.8)
Discontinuation	(*n* = 16) (%)
Neutrophil count	5 (26.3)
> 1.0 × 10^9^/L	3 (60.0)
> 2.0 × 10^9^/L	1 (20.0)
Within normal range	1 (20.0)
Discharge	2 (10.5)
Individual basis	7 (36.8)
Treatment completion	1 (5.3)
Within 4 weeks of treatment completion	1 (5.3)
Nutritional status influence	(*n* = 13) (%)
Yes, discontinuation	1 (7.7)
Yes, initiation & discontinuation	3 (23.1)
No	9 (69.2)
Who governs ND use	(*n* = 13) (%)
Dietetics	12 (92.3)
Catering service	2 (15.3)
Haematology/Oncology	6 (46.1)
Nurses	3 (23.1)
Discharge diet	(*n* = 12) (%)
Neutropenic diet	0 (0.0)
Food safety diet	7 (58.4)
No recommendation	5 (41.6)

Most dietetics teams (92.3%) were involved in governing ND use in the hospital, typically in collaboration with oncology, haematology, food service, and nursing staff. Of the 12 dietitians whose hospitals no longer prescribe the ND, four reported that it had previously been used. One noted that it was discontinued 5–10 years ago, while the others were unsure of the timing. Four provided reasons for discontinuation, citing changes in the evidence base indicating the ND is no longer best practice (P040), perceiving it as unnecessary (P001), or being unsure of the rationale.

Finally, after reviewing an abstract and link to a recent systematic review on the ND, which concluded that it does not significantly reduce infection or mortality compared with a more liberalised diet such as the FSD [18], dietitians provided free‐text responses to the following question *“Based on your own opinion and knowledge of the evidence base, how do you think we should proceed with the use of neutropenic diets for cancer patients?”* (Table 5). Twenty‐seven dietitians responded (56.3%). Eleven dietitians supported continued use of a ND, all of whom prescribed it in their hospitals. Twelve opposed its use, nine of whom worked in hospitals that did not prescribe it. Key themes included the importance of patient food safety education (*n* = 4), concerns that the ND increases malnutrition risk (*n* = 9), insufficient evidence supporting ND efficacy (*n* = 6), preference for FSDs and hygiene practices (*n* = 13), and the value of patient‐specific dietary restrictions (*n* = 10).

**Table 5:** Opinions on use of the neutropenic diet as reported by hospital dietitians.

Themes	Number (*n* = 27)	Representative responses
Support ND use	11	*“Mandatory neutropenic diets options in hospitals for cancer patients, if applicable.”* (P011)
		*“It should still be continued with patients who have low neutrophil level that are at high risk of food‐related illness until there is a consensus and to be discussed with managing teams”* (P022)
Do not support ND use	12	*“I think that it is largely unnecessary as patients are likely to eat food from home/takeaway if they are heavily restricted in hospital.”* (P026)
		*“I think the elimination of all fresh fruit/veg and undercooked meats etc is extreme, especially given the evidence suggests that it is of minimal benefit and this patient group is already a much higher risk of malnutrition.”* (P047)
Education of patients		
Food safety	4	*“I feel patients should be educated on safe food handling, preparation, storage and transport but should not be required to restrict specific foods…”* (P028)
ND	1	*“More education for staff and patients would be great.”* (P029)
High‐risk foods	3	*“…I do feel that there is still a need to restrict very high risk foods during their neutropenic phase given their lack of immune system.”* (P028)
Malnutrition		
Increased risk	9	*“…Neutropenic diets should not be implemented as it further restricts food options and increasing risk of malnutrition…”* (P038)
No risk	1	*“…The food items we restrict on our inpatient menu for neutropenic patients are minimal, and do not result in an overly restrictive diet. There are plenty of nutritious options available (well cooked meats, washed fruit + veg etc) that are not high risk (soft cheeses etc)…”* (P037)
Evidence		
Lack of evidence	6	*“…If there is evidence showing the use of a neutropenic diet does not reduce the rate of infection, we need to ask ourselves why this is still standard practice…”* (P041)
More evidence required	1	*“I think there needs to be agreed‐upon definitions and foods included which will allow more specific studies to be completed to see if there is any supporting evidence.”* (P020)
Diet/restrictions		
Food safety diet	13	*“… general safe food handling advice should be given to patients.”* (P036)
		*“…I think general food safety rules should apply for all patients with avoidance of high risk foods only when absolutely necessary (i.e. food safety or security is a concern, I would not advise consumption of raw fish/meat)…”* (P047)
Patient specific diet	10	*“…I believe it should be used on an individual basis and not for every patient.”* (P047)
		*“Only should be used in the very immunocompromised (e.g. stem cell transplant patients) and should be discontinued when neutrophils begin to rise… It should not be used in general oncology patients, rather education re high risk foods should be provided.”* P010)
Based on neutrophil count	6	*“It should still be continued with patients who have low neutrophil level that are at high risk of food‐related illness until there is a consensus and to be discussed with managing teams.”* (P022)
Limit high‐risk foods	4	*“…We feel the risk‐benefit equation is still in favour of restricting some food items known to carry a higher risk of salmonella, listeria, e‐coli etc food poisoning.”* (P037)
Low‐listeria diet	3	*“…I think general food safety guidelines and listeria guidelines provide enough protection.”* (P017)
Short duration of ND	2	*“…should be discontinued when neutrophils begin to rise. It should not be a long term diet given how restrictive it is limiting food options in a patient group that are already at high risk of malnutrition…”* (P010)
Consensus of recommendations	4	*“Need to have consensus on the recommendations we provide to patients…”* (P018)
Difficult cancer journey	1	*“…This patient population already has enough to worry about, multiple dietary restrictions can be overwhelming and make their cancer journey harder than it needs to be.”* (P040)
Ingrained dogma	1	*“…I think there should be a profession wide shift away from a neutropenic diet if there is no evidence backing it up. I believe it will be difficult to move away from the mindset of patients requiring a neutropenic diet as this appears to be so ingrained into dietitians, particularly those who have been practicing for a number of years.”* (P041)
Clinical practice in other hospitals	1	*“…It would be interesting to see what other institutions are following in terms of defining what foods are high risk (thus not allowed on neutropenic diet), and what constitutes an immunocompromised patient…”* (P037)

## 4 Discussion

To our knowledge, this is the first national survey of Australian dietitians examining hospital dietary practices and restrictions for patients with cancer. We found that over half (56%) of surveyed hospitals continue to prescribe the ND consistent with reports from Europe [10, 11, 12, 13], the United States [14] and China [15] where reported rates of ND usage remain between 50% and 80%. This embedded clinical practice remains despite evidence from ESPEN guidelines and systematic reviews concluding that the ND confers no benefit over a FSD [5, 6, 7, 18, 19, 20, 21, 22, 23].

The body of evidence now reinforces the lack of clinical benefit associated with ND when compared directly with FSD. Multiple systematic reviews and meta‐analyses have consistently demonstrated that the ND does not reduce rates of infection, bacteraemia, febrile neutropenia, or mortality among oncology or haematology patients undergoing immunosuppressive therapy [18, 19, 20, 21, 22, 23]. Similarly, randomised and observational studies included in these reviews show no advantage of excluding raw fruits and vegetables or other commonly restricted foods when appropriate food hygiene practices are applied [24, 25, 26]. The more liberalised FSD approaches, focused on safe food handling, storage, and avoidance of only a small number of high‐risk items, have been shown to provide comparable safety while supporting greater dietary variety and potentially reducing the risk of malnutrition. This evidence base underpins the ESPEN guideline recommendations that specifically advise against the use of the ND in cancer care and instead endorse standard or food‐safety–based diets as safe and appropriate alternatives [5, 6, 7]. Together, these findings highlight that it is the principles of food safety, rather than stringent dietary restriction, that underpin effective risk reduction for immunocompromised patients.

The continued use of the ND reflects a tension between evidence and practice, with 25% of dietitians in our study advocating for replacing the ND with an FSD, which is less restrictive and emphasises safe food handling, and permits greater dietary variety [4]. Conversely, 23% of participants justified ND use on the basis of immunocompromised patient, highlighting the perceived risk of infection despite contradictory evidence. Opinions were closely aligned with institutional practice, with 87% of dietitians reporting views consistent with their hospital's approach, reflecting the strong influence of hospital policies and governance. Similar barriers to change have been observed internationally, suggesting that institutional norms, rather than evidence, continue to drive practice [27].

Hospital type and specialty also shaped ND use. Larger hospitals and those providing haematopoietic stem cell transplants (HSCTs) were more likely to prescribe NDs, likely reflecting historical practices such as sterile diets, laminar flow rooms, and gut decontamination. [2, 28, 29] Restricted foods in Australian hospitals, such as unpasteurised dairy, soft cheeses, undercooked red meat, raw/partially cooked fish and shellfish, and pâté, were broadly consistent with those reported in the United Kingdom [12] and the United States [14] although some differences were noted. For example, deli meats were restricted by most Australian dietitians, but were permitted by many US paediatric oncologists [14]. Conversely, berries and raw honey were less frequently restricted in Australia compared with US practice [14]. Flexibility was evident in policies on takeaway foods and meals brought from home. Most dietitians permitted takeaway food (83.3% ND and 100% non‐ND hospitals), although some restricted raw or high‐risk items, consistent with the United Kingdom [12]. Comparable US data showed mixed restrictions on takeaway and fast food [14]. Nearly all Australian dietitians allowed home‐brought foods, usually with restrictions, consistent with findings from the United States [15]. Such flexibility may improve dietary intake and patient satisfaction [2, 30].

Nutritional status rarely informed decisions to initiate or discontinue ND in this survey, despite being a strong predictor of cancer outcomes [31]. In this survey, all dietitians reported that patients were screened; however, nutritional status did not influence the decision to initiate or discontinue the ND for the majority (≈70%) of dietitians. Instead, initiation was most commonly based on neutrophil count (42.1%), although the specific threshold used varied across respondents. This variability suggests a lack of consensus and highlights the absence of standardised criteria for ND use in practice. Comparable findings were reported in the United States, where most paediatric oncologists also relied on neutrophil count as the primary trigger for ND initiation in oncology patients, while HSCT recipients were more likely to commence the ND at admission or treatment preparation, irrespective of neutrophil levels [14]. These findings underscore the need for clearer evidence‐based guidelines, as reliance on neutrophil count alone may not fully capture infection risk or nutritional needs.

Food safety education was inconsistently delivered. While NEMO resources [17] were most commonly used, provision varied across hospitals, and additional guidance often focused narrowly on food avoidance. Evidence suggests that patients value tailored, structured dietary education [32], underscoring the need for systematic, evidence‐based resources that emphasise safe practices without excessive restriction.

A recurring theme was the need for continuing professional education. Some dietitians acknowledged limited awareness of the literature, while others emphasised the challenge of shifting away from ingrained ND practices in hospitals, and for those with many years of clinical experience. Similar findings have been reported in qualitative studies with oncology dietitians [33]. This tension suggests that while education is critical, institutional and cultural barriers must also be addressed to enable meaningful change.

A key strength of this study is its novelty: it represents the first comprehensive survey of ND practices in Australian hospitals, providing critical baseline data. The purpose‐built survey allowed tailored exploration of current practice. However, limitations include a low response rate (43 responses from 149 hospitals), partly reflecting competing demands during COVID‐19. To minimise respondent burden, the survey did not capture the rationale underpinning dietary decisions, limiting insight into the drivers of practice. As responses were self‐reported, it remains unclear whether they reflected individual practice or hospital protocols. Missing data were common for open‐ended items, and the survey tool was not validated, limiting certainty in reliability.

This survey demonstrates that the ND remains widely used in Australian hospitals, as well its variability in use, despite evidence and international guidelines advising against its use. While some dietitians expressed willingness to move toward more liberalised diets such as the FSD, which is backed by evidence, systemic change will require multidisciplinary collaboration and institutional commitment. Additionally, future clinical guidelines may benefit from clearer recommendations regarding nutrition, including whether guidance should focus on general food safety principles rather than the restrictive ND. Furthermore, continuing education for dietitians is critical to ensure evidence‐based practice, alongside development of tailored patient education resources that emphasise food safety without unnecessary restriction. Broader reviews of current practices and barriers to change are needed to understand why the ND persists and to guide strategies for aligning hospital dietary protocols with contemporary evidence. This study provides a crucial baseline of ND use in Australian hospitals, informing targeted interventions, guideline development, and future research to support evidence‐based dietary practices for patients with cancer.

## Author Contributions

**Trinity Gulliver:** conceptualisation, methodology, survey development, formal analysis, investigation, data curation, writing – original draft, writing – review and editing, visualisation, project administration. **Melissa Hewett:** survey development, writing – review and editing. **Panagiotis Konstantopoulos:** survey development, writing – review and editing. **Lisa Tran:** survey development, writing – review and editing. **Evangeline Mantzioris:** conceptualisation, methodology, survey development, validation, investigation, data curation, writing – review and editing, visualisation, project administration.

## Funding

The authors received no specific funding for this work.

## Conflicts of Interest

The authors declare no conflicts of interest.

## Data Availability

The data that support the findings of this study are available on request from the corresponding author. The data are not publicly available due to privacy or ethical restrictions.
